# Growth and Volatile Organic Compound Production of *Pseudomonas* Fish Spoiler Strains on Fish Juice Agar Model Substrate at Different Temperatures

**DOI:** 10.3390/microorganisms11010189

**Published:** 2023-01-12

**Authors:** Foteini F. Parlapani, Dimitrios A. Anagnostopoulos, Evangelia Karamani, Athanasios Mallouchos, Serkos A. Haroutounian, Ioannis S. Boziaris

**Affiliations:** 1 Laboratory of Marketing and Technology of Aquatic Products and Foods, Department of Ichthyology and Aquatic Environment, School of Agricultural Sciences, University of Thessaly, Fytokou Street, 38446 Volos, Greece; 2 Laboratory of Food Chemistry and Analysis, Department of Food Science and Human Nutrition, Agricultural University of Athens, Iera Odos 75, 11855 Athens, Greece; 3 Department of Animal Science and Aquaculture, Agricultural University of Athens, Iera Odos 75, 11855 Athens, Greece

**Keywords:** fish, spoilage, *Pseudomonas*, metabolism, VOC

## Abstract

Microbial spoilage is the main cause of quality deterioration in seafood. Several strains of psychotropic *Pseudomonas* have been found to dominate in such products, producing a plethora of volatile organic compounds (VOC). Herein, we investigated the growth of and VOC production by seven strains of *Pseudomonas* associated with spoiled fish after inoculation as single and mixed cultures on model fish substrate and storage at 0, 4 and 8 °C. The results indicated a strain-dependent VOC profile that was also affected by the storage temperature. Hierarchical cluster analysis (HCA) successfully grouped the strains based on VOC profile at each studied temperature, while some potential Chemical Spoilage Indices (CSI) were revealed. The findings of the present work will contribute to the understanding of the metabolic activity of particular strains of *Pseudomonas* and to reveal any potential CSI for rapid evaluation of fish spoilage/freshness status.

## 1. Introduction

Microbial spoilage is responsible for undesirable changes in the sensory attributes of fish and fishery products. Microorganisms, in particular so-called Specific Spoilage Organisms (SSO), grow faster than others during storage and cause the formation of metabolites with unpleasant off-odors and off-flavors, leading to the sensory rejection of the product [1,2]. The physiology of microbial cells (e.g., physical and chemical requirements, competitive behavior) and the availability of glucose and other nutrients (e.g., lactate and amino acids in fish flesh (energy sources for microbial growth and metabolism)) are the main factors that determine the dominant microbial association and consequently the type of metabolic compounds in the product under particular storage conditions [2,3,4].

At the genus level, *Pseudomonas* has been found to constitute the main spoiler of aerobically stored fish [2]. Meanwhile, within the genus of *Pseudomonas*, different species or strains e.g., *P. fragi*, *P. putida*, *P. fluorescens*, *P. lundensis*, *P. gessardii* have alternatively been found to dominate in the products depending on factors (e.g., intrinsic, extrinsic, processing, and implicit), which differ from case to case [5,6,7,8,9]. Fish origin, batch-to-batch or lot-to-lot variability, and the type of product (e.g., whole, gutted, filleted) also determine the selection of microbial association at strain level in fish each time [10,11,12]. Thus, spoilage depends on an even smaller fraction of SSO, the so-called Ephemeral Spoilage Organisms (ESOs), which are the result of factors that dynamically persist or are imposed post harvest or post farm gate (e.g., during handling, processing, transportation, storage, etc.) [13]. In this respect, *Pseudomonas* strains seem to be the ESOs of aerobically stored fish from temperate waters.

Microorganisms consume the available nutrients of fish tissue and produce metabolites such as alcohols, aldehydes, ketones, organic acids, sulphides, esters and amines. Such compounds could be used as markers of freshness/spoilage status, known as Chemical Spoilage Indices (CSI) [1,3]. However, a suitable CSI for the assessment of microbial spoilage of fish should (a) be a metabolite produced by the dominant spoilage microorganisms, (b) be absent or at least present at low levels at the beginning of shelf life, and (c) increase during storage, showing a good correlation with microbial growth (in abundance and population) and sensory score [14]. In the literature, there are publications confirming that different bacterial genera can produce different volatile organic compounds (VOCs) through their metabolism and as a result can cause a different type of spoilage each time [2,3,15]. Although there are studies indicating the link of particular species of *Pseudomonas* with the production of particular compounds (e.g., *P. fragi* with ethyl and methyl esters [16,17,18,19]), this is not clear for various strains of *Pseudomonas* dominating in fish and fishery products during storage.

The aim of this work was (i) to record and compare the growth and VOC production of seven strains of *Pseudomonas* isolated from spoiled fish, and (ii) to elucidate the VOCs produced by each strain in single cultures and record their tendency to increase or not in single and mixed cultures in model fish substrates stored at various chilled temperatures. The results will give insights into the spoilage potential and activity of *Pseudomonas* fish spoilers on a model fish substrate and offer information for revealing potential CSI of aerobically stored fish.

## 2. Materials and Methods

### 2.1. Origin of Bacterial Strains

A total of seven (7) *Pseudomonas* strains were used. The strains were previously isolated from chill-stored whole, filleted and gutted sea bream (*Sparus aurata*) at the end of shelf life and identified using 16 S rRNA gene sequence analysis (Table 1). Furthermore, they were stored in the microbial collection of the Laboratory of Marketing and Technology of Aquatic Products and Foods, in frozen beads (Protect, UK) at −80 °C. For the experiment, the strains were resuscitated twice in TSB (Tryptone Soy Broth) after incubation at 25 °C for 48 h, followed by centrifugation at 1500 g for 10 min. The obtained pellets were washed twice and stored at −20 °C until further use. Before inoculation on the model substrate, the pellets were resuspended in sterile saline (0.85% *w*/*v* NaCl).

### 2.2. Preparation, Inoculation and Storage of Model Substrate

Sterile fish juice agar (FJA) model substrates were prepared as described previously by Parlapani et al. [15]. For the inoculation, the initial population for all strains used was adjusted to 3 logs cfu/mL (confirmed by plate counting). The inocula for each of the 7 strains, as well as a mix of them, was spread on the surface of the FJA. A non-inoculated FJA (control) was used to determine the production of non-microbial-origin VOCs. The models were stored at 0, 4 and 8 °C (two proper and one abuse storage temperatures for fish products) for 20, 10 and 4 days, respectively.

### 2.3. Enumeration of Bacterial Population

Microbiological analysis was carried out every 48 h, 24 h and 12 h for the models at 0, 4 and 8 °C, respectively. More specifically, 1 g from each FJA was added to 9 mL sterile MRD (Maximum Recovery Diluent, 0.85% NaCl), followed by vortex for 2 min. A volume of 0.1 mL was spread on TSA (Tryptone Soy Agar, LAB M, Lancashire, UK) and incubated at 25 °C for 48 h. Results were expressed as mean log cfu/g ± standard deviation of 3 replicates.

### 2.4. Determination of Volatile Organic Compounds Profile

Headspace Solid Phase Micro-Extraction (SPME) method was used for the extraction of the volatile compounds as described previously by Parlapani et al. [15]. An amount of 10 g of FJA substrate was removed from each of the three different FJA plates for each treatment (single or mixed culture) and pooled. Then, 2.5 g of the pooled sample in duplicates were transferred into a 20 mL glass vial, which was closed hermetically using a mininert valve (Sigma Aldrich, Argyroupoli, Greece), and the contents were left for 15 min at 40 °C. After that, under the same conditions, the SPME fiber (DVB/CAR/PDMS 50/30 mm) was exposed to the headspace for another 30 min. The length of the fiber in the headspace was kept constant. Before the analysis, the fiber was exposed to the injection port held at 250 °C for 10 min. VOC separation and identification were performed according to Parlapani et al. [21], and the results expressed in arbitrary units of the peak area of deconvoluted component multiplied by 10^−6^ in order to monitor their tendency to increase or not during storage.

### 2.5. Modeling and Statistical Analysis

The microbial population changes were fitted using the Baranyi equation [22] $y(t)=y_{\max}-\ln[1+(e^{-y_{\max}-y_{0}}-1)e^{\mu_{m}A_{n}(t)}]$, where *y*(*t*) is the logarithm of population at time t, *y*_max_ is the logarithm of maximum population, *y*_0_ is the logarithm of initial population, *μ_m_* is the maximum specific growth rate, and *A_n_*(*t*) is a function related to the physiological state of the cells. DMFIT software (Institute of Food Research, Reading, UK) was used for fitting and growth rate estimation.

Differences between means in bacterial populations and kinetic parameters were statistically tested by performing Analysis of Variance (one-way ANOVA) followed by LSD (Least Significant Difference) test (*p* ≤ 0.05), using the IBM^®^ SPSS^®^ statistics 20 software (SPSS Inc., Chicago, IL, U.S.). Furthermore, to evaluate potential relationships between strains and the VOC produced during storage, a Hierarchical Cluster Analysis (HCA) was performed based on Euclidean distance and Ward’s linkage for similarity measure and clustering algorithm, respectively. Prior to analysis, data were log-transformed and pareto-scaled, and the results presented in the form of heatmaps. The analysis was applied using the Metaboanalyst 5.0 platform [23].

## 3. Results

### 3.1. Microbial Populations and Kinetics

The microbial population changes and kinetic parameters of *Pseudomonas* in FJA substrate during storage at 0, 4 and 8 °C are presented in Figure S1 and Table 2, respectively. The initial populations were adjusted and varied from 2.78 ± 0.22 to 2.96 ± 0.23 log cfu/g (*p* > 0.05). At every storage temperature, several fluctuations were observed in strain populations at the middle stage of storage, exhibiting, in many cases, significant differences from each other, indicating a strain-dependent growth ability. However, at the end of storage most of the strains exhibited similar population levels without significant differences (*p* > 0.05). At 0 °C, the highest population levels were recorded for strains 55 (Bacterium SBF-B18-dsp: *P. fragi*) and 35 (Bacterium SBF-B5-d0: *P. fluorescens*) at D 20, followed by the strains 83 (*Pseudomonas* sp. GSB-b11), 63 (*Pseudomonas fragi* strain b67), 61 (Bacterium SBF-A12-dsp: *P. fragi*) and 45 (Bacterium SBF-B19-dsp: *P. fragi*) (Table 2). In the mixed cultures, the population reached the levels of 9.19 ± 0.05 log cfu/g (*y*_max_ = 9.13 ± 0.05), indicating similar population level compared to that of the aforementioned strains. At 4 °C, the strains 55, 45, 35 and 61 reached the highest population levels than others; however, their populations were not statistically different (*p* > 0.05) from those of the mixed culture (9.54 ± 0.17 log cfu/g, *y*_max_ = 9.55 ± 0.07) or of the rest of the strains (Table 2). At 8 °C, the strains 83, 61 and 71 (*P. fluorescens* strain b84) reached the highest population levels than other strains and differences in population levels were recorded in some cases (Table 2).

Lag phase (duration in days) was observed for almost all strains in models at every studied temperature, apart from 35 at 0 °C, 55 and 83 at 4 °C and 71 at 8 °C (Table 2). Regarding the maximum specific growth rate (*μ_m_* or *μ_max_*), strain 63 (followed by 45 and 71) grew faster (*μ_max_* = 0.775 ± 0.010) than the others at 0 °C, while strain 83 was the slowest (*μ_max_* = 0.484 ± 0.044). At 4 °C, strain 63 also presented the highest maximum specific growth rate (*μ_max_* = 1.208 ± 0.081), while strain 83 was again the slowest of the *Pseudomonas* strains (*μ_max_* = 0.733 ± 0.044). At the abuse storage temperature (8 °C), strain 45 was the fastest of the *Pseudomonas* strains in FJA models (*μ_max_* = 1.872 ± 0.211), followed by strain 55 (*μ_max_* = 1.653 ± 0.317), while once again, strain 83 presented the lowest maximum specific growth rate (*μ_max_* = 1.374 ± 0.140) compared with the rest of the studied strains (Table 2).

### 3.2. VOC Profile and Correlation Analysis

Τhe detected VOCs throughout storage at 0, 4 and 8 °C mainly consisted of esters, alcohols, ketones and aldehydes, while the presence of other compounds (e.g., limonene, ethane, 1,2-diethoxy and n-nonane) was also noticeable (Table S1). A total of 92, 60 and 78 VOCs were detected at temperatures of 0, 4 and 8 °C, respectively (without taking into account terpenes, aromatic compounds and hydrocarbons that were found in trace amounts). Several—56, 26 and 41 VOCs at 0, 4 and 8 °C, respectively—were produced in inoculated models only (e.g., methanethiol, 2-methyl-1-butanol, isopropyl alcohol, dimethyl sulfide, dimethyl disulfide, 2-methylbutyraldehyde, 3-methylbutyraldehyde, 2-pentanone, 2-heptanone, n-nonanone, 2-undecanone, 2-nonanone, 3-pentanone, butyl butyrate, isobutyl isobutyrate, ethyl-2 methylbutyrate, ethyl isovalerate, ethyl isobutyrate, ethyl butyrate, ethyl acetate, ethyl octanoate, ethyl propionate, ethyl tiglate, lauric aldehyde, etc.). The rest of the VOCs (36, 34 and 37 at 0, 4 and 8 °C, respectively) were found in both inoculated and non-inoculated models (e.g., 2-ethyl-1-hexanol, 1-penten-3-ol, 2-hexen-1-ol (trans), nonanal, n-decanal, heptanal, octanal, hexenal, 6-methyl-5-hepten-2-one, acetophenone, etc.), respectively (Table S1). At the end of storage, the VOC profiles differed to some extent among the three studied temperatures, while in some cases, it was strain dependent.

**Figure 1:**
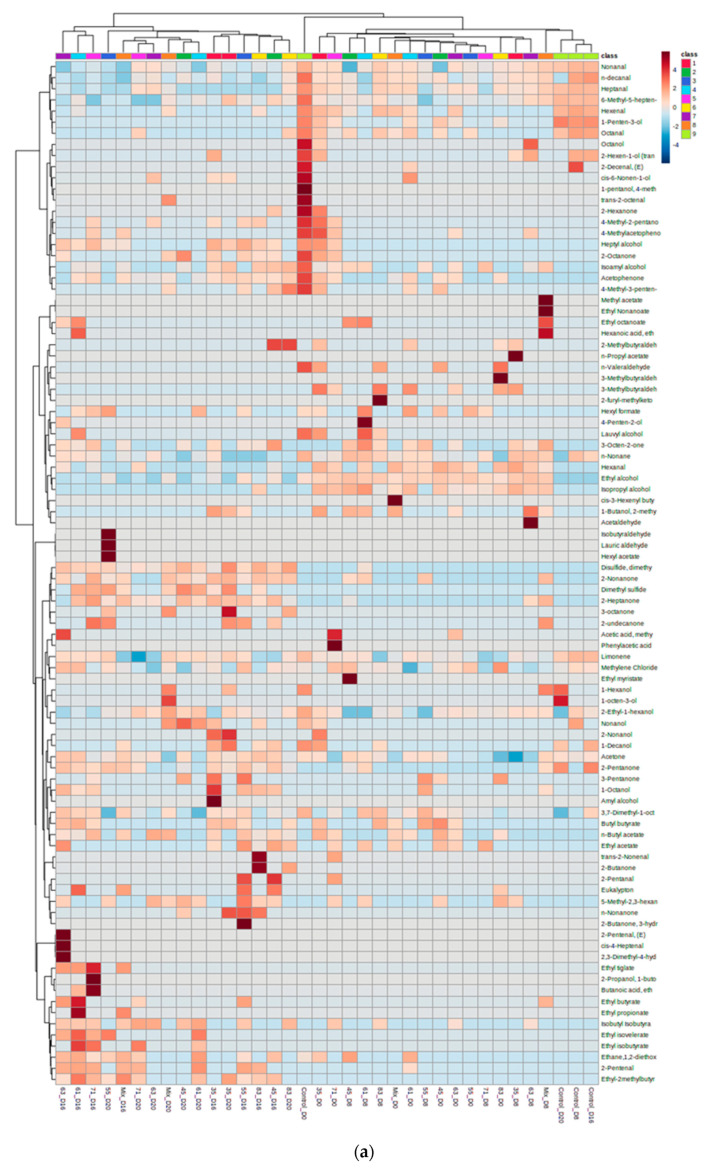

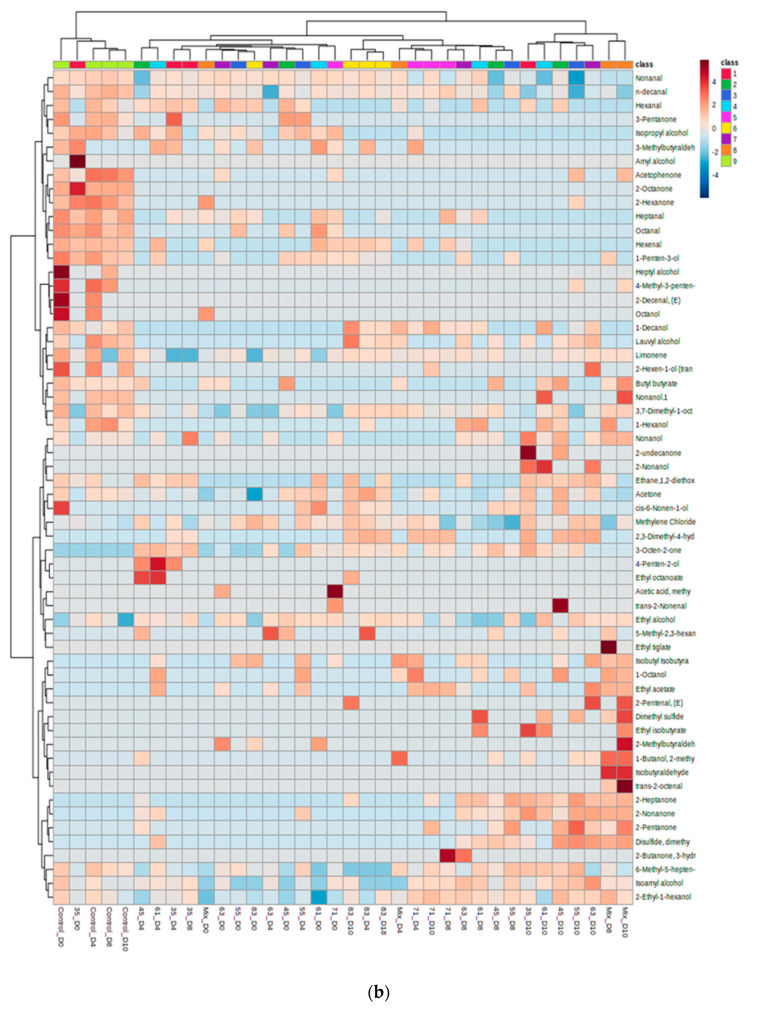

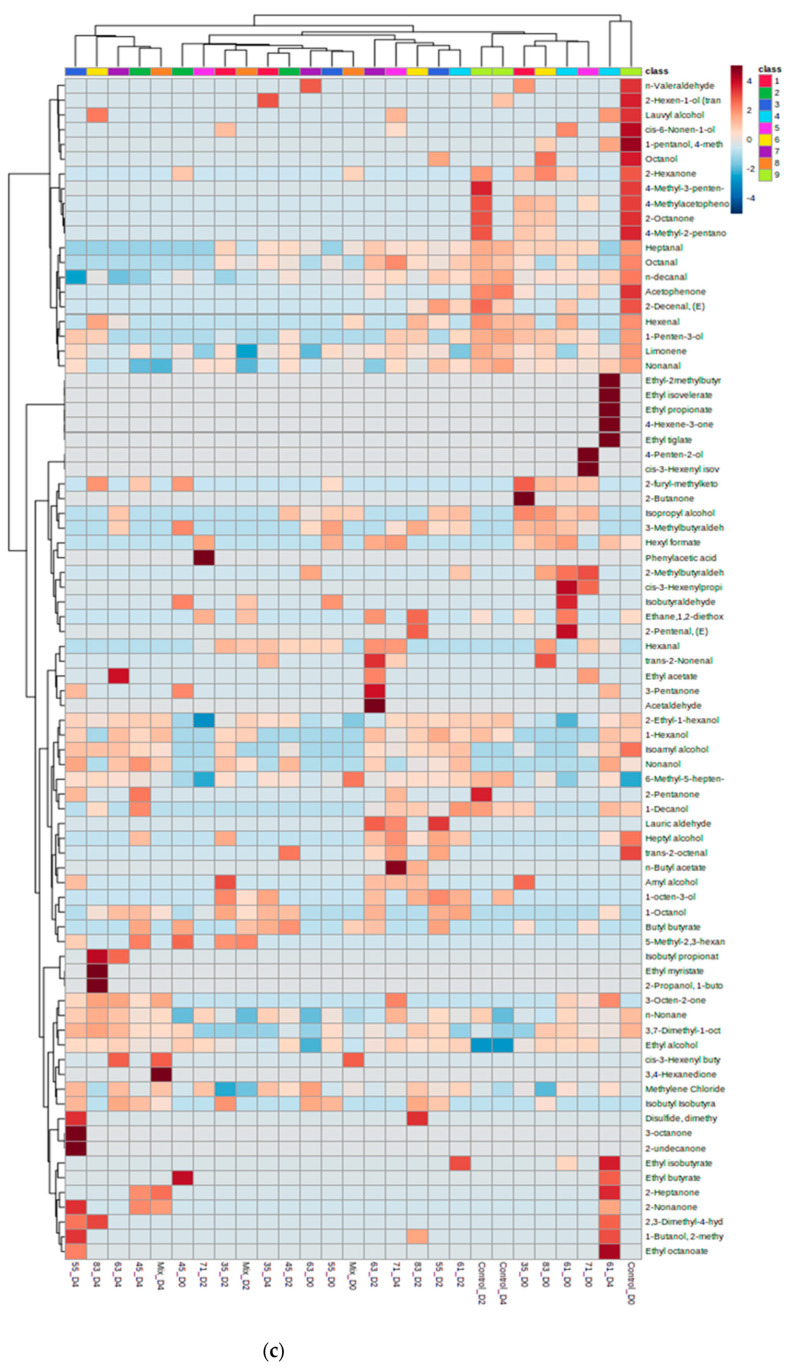
Hierarchical cluster analysis on heatmap plot between strains and VOC profile, during storage of inoculated fish juice agar model substrates at 0 °C (**a**), 4 °C (**b**) and 8 °C (**c**).

According to HCA, a group of VOCs exhibited a stable profile—always detectable and closely related with either initial or final stages of storage—depending on the strain and storage temperature (Figure 1). Another group of VOCs exhibited a quite unstable tendency, since many of them were detectable sporadically at intervals. In the non-inoculated FJA in general, the majority of VOCs that were found initially (D 0) were also closely linked to this treatment throughout the storage period, with specific exceptions (e.g., 1-decanol, n-valeraldehyde and some other alcohols and ketones). Furthermore, there was no compound that was produced solely in the non-inoculated models during the latest stages of storage in any of the studied temperatures.

According to the heatmap plot at 0 °C (Figure 1a), the models could be divided into two main clusters, clearly separated based on storage time. More specifically, the first cluster included both single and co-cultured FJA models at the initial and middle stages of storage. The non-inoculated models, including all storage days, were also a part of this group. The first cluster was mainly linked with the production of alcohols (e.g., ethyl alcohol, isopropyl alcohol, 1-penten-3-ol, 2-hexe-1-ol (trans), etc.), aldehydes (e.g., nonanal, hexenal, heptanal, octanal, hexanal, etc.) and ketones (e.g., 2-octanone, 4-methyl-2-pentanone, etc.). The second clustered group included samples after the middle of storage (D 16 and D 20). The samples belonging to the second group exhibited a different VOC profile, since they were closely related with esters such as ethyl isovalerate, ethyl isobutyrate, isobutyl isobutyrate, ethyl-2-methylbutyrate, dimethyl sulfide, butyl acetate and ketones such as 2- heptanone and 2-nonanone. Apart from those VOCs well-shared between the different treatments, it is crucial to highlight a unique and/or strong correlation profile between specific VOC and strains at D 20. For instance, lauric aldehyde, isobutyraldehyde and hexyl acetate were exclusively linked with strain 55 (Bacterium SBF-B18-dsp: *P. fragi*), while 2-methylbutyraldehyde and 4-methyl-3-penten were solely linked and strongly correlated, respectively, with strain 83 (*Pseudomonas* sp. GSB-b11). Additionally, strain 35 (Bacterium SBF-B5-d0: *P. fluorescens*) was strongly linked with 3-octanone and 2-nonanol, while the mixed culture was the only case linked with 1-octen-3-ol, trans 2-octenal and 1-hexanol at the end of the storage.

With respect to storage at 4 °C (Figure 1b), the models were divided into three main groups. More specifically, the first group included the control treatment throughout storage. This cluster was closely related the production of alcohols (e.g., ethyl alcohol, isopropyl alcohol, 1-penten-3-ol, 2-hexe-1-ol (trans), etc.), aldehydes (e.g., nonanal, octanal, hexanal, etc.), and ketones (e.g., 2-octanone, acetophenone, 2-hexanone etc.). The second group included mixed culture at the latest stages of storage (D 8 and D10). This group produced a specific VOC profile being strongly related to ketones (e.g., 2-heptanone, 2-nonanone, 2-pentanone, etc.) and aldehydes (e.g., 2-methylbutyraldehyde, isobutyraldehyde), while the presence of 1-butanol, 2-methyl was also remarkable. Similarly, the third cluster, constituted mainly by all the remaining cases at D 8 and D 10, was characterized by similar ketone profiles to those in the second group; however, in this case, the production of aldehydes compared to that in the second group was negligible. The latter was the main reason for the separation between the second and the third cluster.

On the other hand, at 8 °C (Figure 1c), there was not a clear separation between the initial (D 0) and the final stage of storage (D 4). In the context of this complexity, a clustered VOC group (i.e., limonene, nonanal, n-decanal, heptanal, 1-penten-3-ol, 2-ethyl-1-hexanol, 1-hexanol, isoamyl alcohol, etc.) exhibited a consistent profile throughout storage in both inoculated and non-inoculated models, while others (i.e, 2-octanone, ethane,1,2-diethoxy, isobutyraldehyde, 4-methyl-2-pentanone, 2-methylbutyraldehyde) were not linked with D 4, in any of the treatments. Furthermore, VOCs including methylene chloride and 2-ethyl-1-hexanol were linked with all storage days in all cases, exhibiting a higher correlation with the last day of storage (D 4). Similar findings were observed for n-nonane, although this compound had no correlation with the non-inoculated treatment at D 4.

Furthermore, in the non-inoculated sample, a group of alcohols and ketones were highly linked with the initial and middle storage stages but not with D 4. A unique and/or strong correlation profile between some VOC and strains was observed at D 4. For example, strain 55 (Bacterium SBF-B18-dsp: *P. fragi*) was strongly correlated with 1-butanol-2-methyl, ethyl octanoate, disulfide dimethyl, 3-octanone and 2-undecanone, while strain 83 (*Pseudomonas* sp. GSB-b11) was strongly related to ethyl myristate and 2-propanol-1-butoxy. In addition, strain 63 (*Pseudomonas fragi* strain b67) was strongly linked with ethyl acetate, strain 35 (Bacterium SBF-B5-d0: *P. fluorescens*) with 2-hexen-1-ol, strain 45 (Bacterium SBF-B19-dsp: *P. fragi*) with 2-pentanone, strain 71 (*P. fluorescens* strain b84) with octanal and n-butyl acetate, while the mixed inoculum was solely related to 3,4 hexanedione. Among the aforementioned treatments (strain 45 and mix) a well-shared ketone group (2-heptanone and 2-nonanone) with high correlation was also observed. Finally, it should be pointed out that strain 63 (*Pseudomonas fragi* strain b67) was solely strongly related to a variety of unique VOCs, including ethyl-2-methylbutyrate, 4-hexene-3-one, ethyl tiglate, ethyl isovalerate and ethyl propionate.

**Figure 2:**
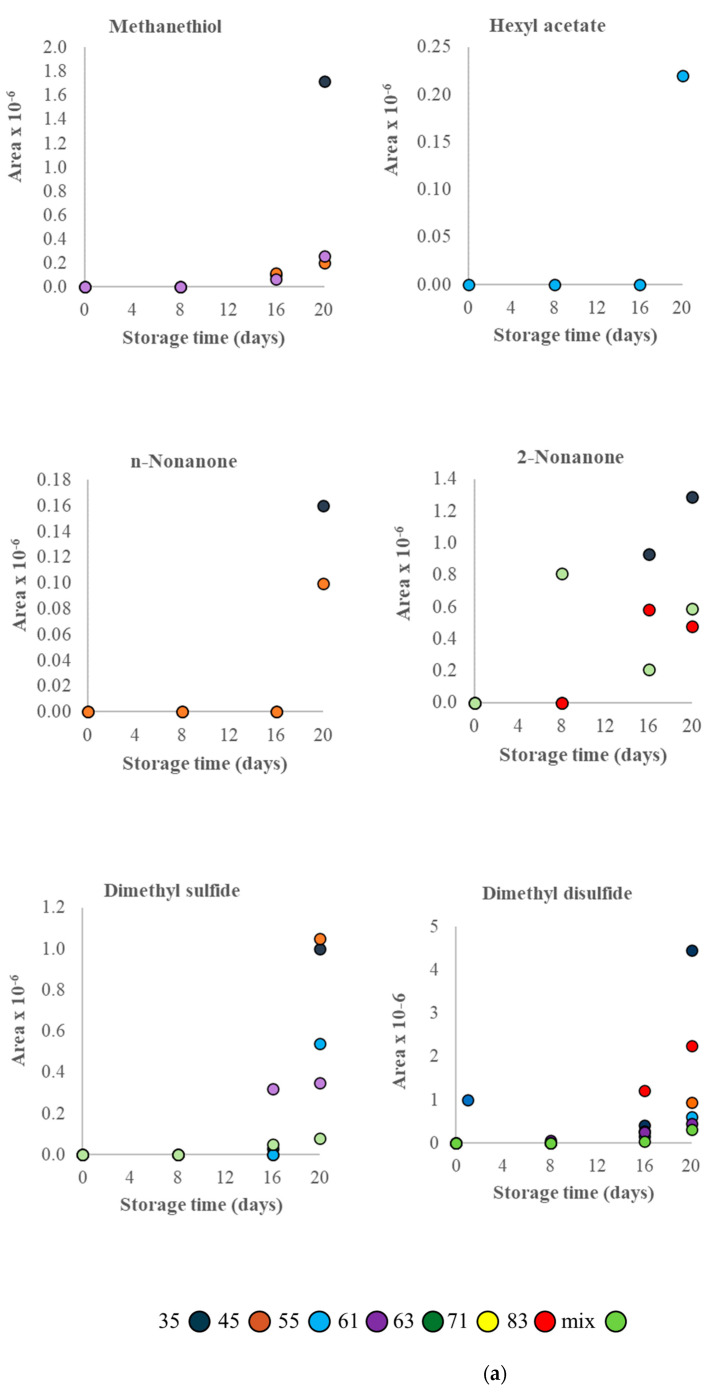

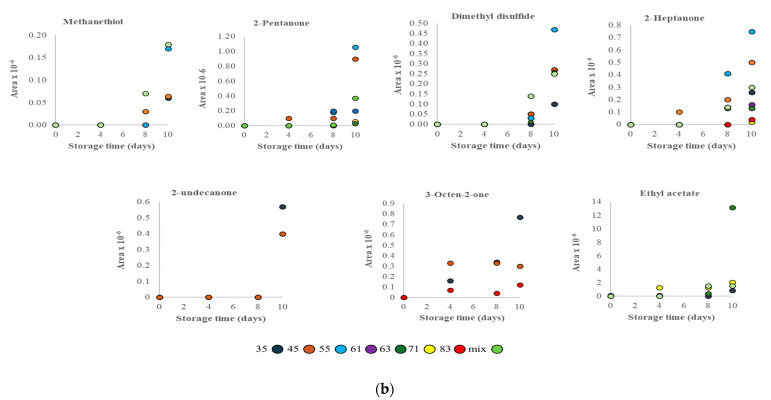

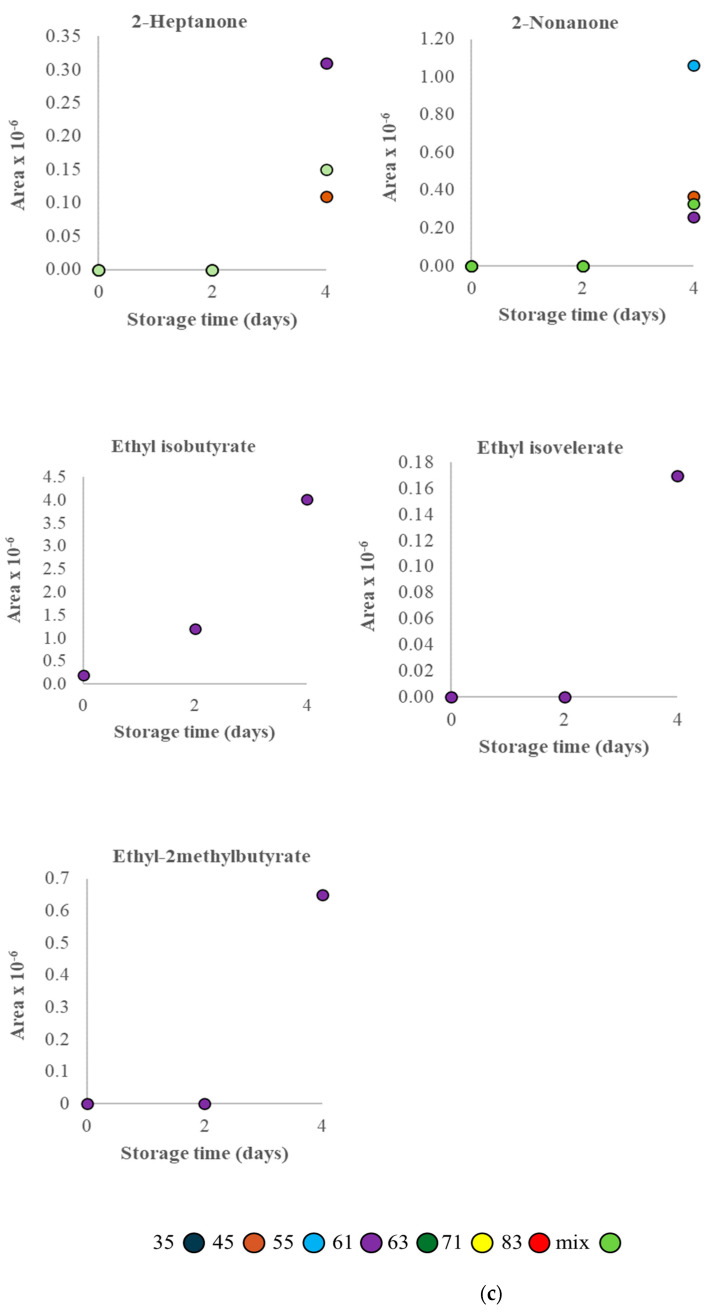
Volatile organic compounds that increased during storage in inoculated FJA model substrates at 0 °C (**a**), 4 °C (**b**) and 8 °C (**c**).

The VOCs that were increased only in inoculated models during storage (Figure 2) possessed a complex and strain-/temperature-dependent profile. At 0 °C (Figure 2a), methanethiol increased during storage in models inoculated with strains 35, 45 and 61, while dimethyl sulfide also increased by the same strains as well as strain 55. An increasing tendency of dimethyl disulfide was also recorded in the inoculated models with each strain (all except 63 and 71) and mix, while n-nonanone was found to increase in those inoculated with strains 35 and 45. Other compounds such as 2-nonanone increased in models inoculated with strains 35, 83 and mix, while hexyl acetate increased only in the model inoculated with strain 55. At 4 °C (Figure 2b), seven compounds were found to increase. Among them, methanethiol increased in models inoculated with strains 35, 45 and 55 and mix, 2-pentanone with 45, 55, 63, 71 and mix, 2-heptanone with each studied strain and mix, dimethyl disulfide with 35, 45, 55, 63 and mix, 2-undecanone with 35 and 45, 3-octen-2-one with 35, 45 and 83, while ethyl acetate with 35, 63, 71 and mix. At 8 °C (Figure 2c), five compounds, in particular ethyl-2methylbutyrate, ethyl isovalerate, ethyl isobutyrate, 2-heptanone and 2-nonanone, were found to increase in models inoculated with strain 61 (the increase in the three esters linked with this strain only); however, 2-heptanone was also found to increase in models with 45 and mix, and 2-nonanone with 45, 55 and mix.

## 4. Discussion

It is well known that strains of psychrotrophic *Pseudomonas* are the main bacteria present in spoiled air-stored seafood originating from temperate waters [2,3]. *Pseudomonas* has been found to cause spoilage to various seafood, including the two most important fish of the Mediterranean aquaculture, the gilt-head sea bream and the European sea bass [5,6,7,8,9]. Different strains of *P. fluorescens* and *P. fragi* (also used in this study) dominated these products in most of cases. Based on our results, regarding the growth of the studied strains in FJA models, a similar profile between all strains in all studied storage conditions (0, 4 and 8 °C) was recorded, since all strains exhibited a remarkable growth rate and population levels in FJA models. In a previous study, Parlapani et al. [15] found a *μ_max_* equal to 0.829 ± 0.032 (d^−1^) for *Pseudomonas* spp. in mixed cultures (composed by other strains, only strain 45 was common in both cases) in FJA models of gilt-head sea bream stored at 0 °C (higher than that recorded in mixed cultures at 0 °C, *μ_max_ =* 0.641 ± 0.071). This finding clearly indicates that different mixes of *Pseudomonas* strains present different growth rates on FJA models under particular storage conditions might be due to interspecies symbiotic or antagonistic microbial interaction. Compared to real fish, the strains inoculated in FJA models presented much higher maximum specific growth rate (*μ_max_*) than those recorded on cetrimide/fucidin/cephaloridine (CFC) agar (a selective culture medium for *Pseudomonas* spp. enumeration) in gilt-head sea bream fillets at similar chilled temperatures [6]. On the other hand, *μ_max_* was found to be much lower than those observed for *Pseudomonas* spp. in gilt-head sea bream at 0, 4 and 8 °C in other studies [24]. The different findings in the aforementioned cases might be due to the domination of different microbial association (including *Pseudomonas* spp.), which is often influenced by microbial interaction in each case [25,26]. Moreover, strains 35 (Bacterium SBF-B5-d0: *P. fluorescens*), 45 (Bacterium SBF-B19-dsp: *P. fragi*) and 55 (Bacterium SBF-B18-dsp: *P. fragi*), isolated from fresh and spoiled sea bream fillets stored at 0 °C [20], also reached higher population levels compared to the others in model systems stored at 0 °C herein, while strains 61 (Bacterium SBF-A12-dsp: *P. fragi*) and 71 (*P. fluorescens* strain b84) isolated from spoiled sea bream stored at higher chilled temperatures [5,20] were also found to reach higher population levels in models stored at 4 and 8 °C. These findings indicate that such model substrates can be exploited as a reliable in vitro tool to monitor the microbial behavior at strain level, being able to successfully simulate real fish flesh. The latter is also in agreement with the findings by Parlapani et al. [15].

The majority of the compounds detected only in inoculated models in the present study (e.g., methanethiol, 2-methyl-1-butanol, isopropyl alcohol, dimethyl sulfide, dimethyl disulfide, 2-methylbutyraldehyde, 3-methylbutyraldehyde, 2-pentanone, 2-heptanone, n-nonanone, 2-undecanone, 2-nonanone, 3-pentanone, butyl butyrate, isobutyl isobutyrate, ethyl-2methylbutyrate, ethyl isovalerate, ethyl isobutyrate, ethyl butyrate, ethyl acetate, ethyl octanoate, ethyl propionate, ethyl tiglate, lauric aldehyde, etc.) have also been found to be produced by various *Pseudomonas* strains in inoculated sterile seafood model substrates, for example, by *P. fragi* in black rockfish at 5, 15, 25 °C [18] and in prawn extract at 5 °C [27], or by *P. fluorescens* in sterile black rockfish at 0 °C [17], confirming that such metabolites come solely from bacterial activity. Additionally, 2- and 3-methyl-1-butanol, ethanol, 2- and 3-methylbutanal, 3-hydroxy-2-butanone, acetic acid, dimethyl sulfide, 3-methylbutyl acetate and various ethyl esters were associated with *Pseudomonas* in FJA models of gilt-head sea bream stored at 0 and 15 °C [15]. Here, it is important to underline the production of such esters by *Pseudomonas* spp. (through esterase activity) in seafood during storage [2]. In real seafood, such VOC were also detected, and increased in some cases, in gilt-head sea bream, European sea bass and meagre, at various chilled storage conditions where *Pseudomonas* spp. dominated [6,7,8,21]. Indeed, many of these compounds can be produced by the catabolism of aminoacids or other compounds of substrate by *Pseudomonas* spp. [13]. On the other hand, the compounds detected in both inoculated and non-inoculated models (e.g., 2-ethyl-1-hexanol, 1-penten-3-ol, 2-hexen-1-ol (trans), nonanal, n-decanal, heptanal, octanal, hexenal, 6-methyl-5-hepten-2-one, acetophenone, etc.) cannot be clearly related to microbial or chemical activity. For example, 2-ethyl-1-hexanol has been found to be linked with the activity of lactic acid bacteria in sterile shrimp [28] and FJA models [15]; however, it has also been reported as a non-microbial origin compound [15]. On the other hand, the rest of the aforementioned compounds, e.g., 1-penten-3-ol, heptanal, nonanal, etc., have already been reported to be products of chemical activity in seafood; in particular, they are involved in the oxidative rancidity of polyunsaturated fatty acids [29,30,31]. Our findings indicate that different storage temperatures affect, to some extent, the metabolic profile of the strains. Indeed, the interactive behavior of microorganisms in seafood not only contributes to their growth, but also their spoilage activity [26].

Some of the compounds of microbial origin presented an increasing tendency during FJA storage, and so might be used as CSIs of the freshness or spoilage status of seafood. Among them, some compounds (e.g., methanethiol, dimethyl sulfide, dimethyl disulfide, 2-pentanone, ethyl acetate, 2-nonanone, n-nonanone, 2-undecanone, 3-octen-2-one, 2-heptanone) were associated with some of the *Pseudomonas* strains (e.g., 35, 35, 55), other compounds were associated with all strains (e.g., 2-heptanone), while others (e.g., hexyl acetate, ethyl-2methylbutyrate, ethyl isovalerate, ethyl isobutyrate) were associated with the action of one strain only (e.g., 61), revealing the spoilage activity of the different strains of *Pseudomonas*. The majority of these compounds were associated with quality deterioration due to the development of off-odors and off-flavors in chill-stored seafood [2]. Indeed, such compounds, mainly the ethyl esters, have also been found to increase in gutted gilt-head sea bream and European sea bass during storage at 2°C where *P. fluorescens* dominated [6,7], and in gilthead sea bream fillets at 0 and/or 5 under air where *Pseudomonas* genus in general dominated [21]. In all these cases, sensory rejection occurred when *Pseudomonas* reached spoilage level (8–9 log cfu/g), and in parallel, some of the aforementioned compounds increased. Taking into account all information and findings, the compounds of microbial origin found herein could indeed be used as potential CSIs in some seafood, such as aerobically stored Mediterranean aquacultured fish.

## 5. Conclusions

Studies using sterile model food systems can give us valuable information on the growth and metabolic potential and activity of microorganisms of interest. Obviously, the growth parameters cannot be the same as those that occur in real food systems due to various implicit factors (e.g., interaction and antagonism between microorganisms, effect of food structure on growth, etc.). However, there is no other way to study microorganisms of the same genus or species; hence, such compromises cannot be avoided. On the other hand, the study of the metabolic activity of single strains on sterile model systems is the only tool for elucidating the compounds produced by those strains. In our study, various VOC were produced in inoculated models only, indicating their microbial origin from single strains, while others were produced in both inoculated and controls indicating the non-microbial origin in most of the cases. Additionally, several VOCs (e.g., methanethiol, dimethyl sulfide, dimethyl disulfide, 2-pentanone, ethyl acetate, 2-nonanone, n-nonanone, 2-undecanone, 3-octen-2-one, 2-heptanone, 2-heptanone, hexyl acetate, ethyl-2methylbutyrate, ethyl isovalerate, ethyl isobutyrate) were found to increase during storage depending on strain and/or temperature. Such compounds are proposed herein as CSI in seafood. Certainly, the metabolic activity might again be affected in complex food systems in which other microorganisms are also present, but in such cases a comparison with other studies on real foods can give us insights on food spoilage mechanisms.

## Figures and Tables

**Table 1:** The bacterial strains used to inoculate the fish juice agar model substrates.

Strain	GenBank Accession Number	Strain	Origin	Reference
35	KJ411280	Bacterium SBF-B5-d0 (*Pseudomonas fluorescens*)	Sea bream fillets stored at 0 °C	[20]
45	KJ411285	Bacterium SBF-B19-dsp (*Pseudomonas fragi*)	Sea bream fillets stored at 0 °C	[20]
55	KJ411284	Bacterium SBF-B18-dsp (*Pseudomonas fragi*)	Sea bream fillets stored at 0 °C	[20]
61	KJ411289	Bacterium SBF-A12-dsp (*Pseudomonas fragi*)	Sea bream fillets stored at 5 °C	[20]
63	KR778800	*Pseudomonas fragi* strain b67	Whole sea bream stored at 0 and 5 °C	[5]
71	KR778803	*Pseudomonas fluorescens* strain b84	Whole sea bream stored at 5 and 15 °C	[5]
83	KP010026	*Pseudomonas* sp. GSB-b11	Gutted sea bream stored at 2 °C	[6]

**Table 2:** Predicted, initial (*y*_0_), final population (*y*_max_), lag phase duration (*lag*) and maximum specific growth rates (*μ_max_*) of *Pseudomonas* strains inoculated in fish juice agar model substrates stored at 0, 4 and 8 °C, as estimated by Baranyi equation (Baranyi and Roberts, 1994). The values in parentheses are the observed initial and maximum population levels.

T °C	Culture	*y*_0_ (log cfu/g)	*y*_max_ (log cfu/g)	*lag* (days)	*μ_max_* (day ^−1^)
0 °C	35	2.85 ± 0.18 (2.78 ± 0.22)	9.52 ± 0.05 ^e^ (9.49 ± 0.09)	0.00	0.515 ± 0.015 ^b^
	45	2.86 ± 0.65 (2.86 ± 0.83)	9.40 ± 0.07 ^c,d,e^ (9.44 ± 0.13)	2.22 ± 0.80	0.755 ± 0.070 ^c^
	55	2.83 ± 0.12 (2.90 ± 0.00)	9.46 ± 0.08 ^d.e^ (9.51 ± 0.07)	1.21 ± 0.47	0.650 ± 0.087 ^b,c^
	61	2.72 ± 0.12 (2.85 ± 0.17)	9.24 ± 0.10 ^b,c^ (9.34 ± 0.08)	1.54 ± 0.02	0.572 ± 0.065 ^b,c^
	71	3.01 ± 0.14 (2.90 ± 0.05)	9.11 ± 0.02 ^b^ (9.12 ± 0.06)	1.50 ± 0.98	0.740 ± 0.155 ^c^
	83	2.66 ± 0.29 (2.79 ± 0.20)	9.29 ± 0.03 ^b,c,d^ (9.23 ± 0.08)	0.76 ± 0.00	0.484 ± 0.044 ^b^
	63	2.88 ± 0.20 (2.79 ± 0.26)	9.22 ± 0.09 ^b,c^ (9.25 ± 0.22)	3.65 ± 0.13	0.775 ± 0.010 ^c^
	Mix	2.89 ± 0.28 (2.96 ± 0.23)	9.13 ± 0.05 ^b^ (9.19 ± 0.04)	1.41 ± 0.15	0.641 ± 0.071 ^b,c^
4 °C	35	3.04 ± 0.13 (2.79 ± 0.22)	9.32 ± 0.09 (9.27 ± 0.12)	1.33 ± 0.09	1.083 ± 0.038 ^e^
	45	2.89 ± 0.42 (2.86 ± 0.34)	9.40 ± 0.13 (9.41 ± 0.15)	0.40 ± 0.70	0.914 ± 0.060 ^c,d^
	55	2.82 ± 0.06 (2.90 ± 0.01)	9.57 ± 0.13 (9.47 ± 0.20)	0.0	0.896 ± 0.048 ^b,c^
	61	2.85 ± 0.22 (2.85 ± 0.17)	9.28 ± 012 (9.32 ± 0.01)	0.67 ± 0.69	0.985 ± 0.158 ^d^
	71	2.79 ± 0.12 (2.90 ± 0.05)	8.76 ± 0.33 (8.73 ± 0.19)	0.11 ± 0.19	0.967 ± 0.055 ^d^
	83	2.68 ± 0.09 (2.79 ± 0.21)	9.46 ± 0.26 (9.24 ± 0.21)	0	0.733 ± 0.044 ^b^
	63	2.89 ± 0.36 (2.79 ± 0.26)	9.08 ± 0.18 (9.21 ± 0.12)	1.10 ± 0.47	1.208 ± 0.081 ^e,f^
	Mix	2.92 ± 0.42 (2.96 ± 0.24)	9.55 ± 0.07 (9.54 ± 0.17)	0.93 ± 0.46	1.040 ± 0.044 ^e^
8 °C	35	2.93 ± 0.27 (2.78 ± 0.22)	8.33 ± 0.32 ^b^ (8.50 ± 0.44)	1.22 ± 0.23	1.434 ± 0.110 ^b,c^
	45	2.93 ± 0.95 (2.86 ± 033)	8.63 ± 0.18 ^b,c^ (8.90 ± 0.03)	0.59 ± 0.19	1.872 ± 0.211 ^c^
	55	2.85 ± 017 (2.90 ± 0.00)	8.69 ± 026 ^c^ (8.83 ± 010)	0.74 ± 0.00	1.653 ± 0.317 ^b,c^
	61	2.84 ± 0.40 (2.85 ± 0.17)	9.16 ± 0.16 ^c,d^ (9.03 ± 0.08)	0.65 ± 0.53	1.492 ± 0.213 ^b,c^
	71	2.83 ± 0.04 (2.90 ± 0.05)	9.07 ± 0.22 ^c,d^ (9.00 ± 0.08)	0	1.409 ± 0.012 ^b^
	83	2.79 ± 0.20 (2.79 ± 0.20)	9.22 ± 0.29 ^c,d^ (9.06 ± 0.02)	0.46 ± 0.35	1.374 ± 0.140 ^a,b^
	63	2.73 ± 0.38 (2.79 ± 026)	8.10 ± 0.21 ^b^ (8.22 ± 0.21)	0.60 ± 0.21	1.473 ± 0.034 ^b,c^
	Mix	2.88 ± 0.29 (2.96 ± 0.23)	9.34 ± 0.19 ^d^ (9.14 ± 0.02)	0.46 ± 0.00	1.435 ± 0.056 ^b,c^

Different letters indicate significant difference (*p* ≤ 0.05).

## Data Availability

The datasets generated for this study are available on request to the corresponding author.

## Supplementary Materials

The following supporting information can be downloaded at: https://www.mdpi.com/article/10.3390/microorganisms11010189/s1, Table S1. VOC produced by seven *Pseudomonas* strains in single and mixed cultures in fish juice agar model substrates at 0 °C (a), 4 °C (b) and 8 °C (c). Control is also presented. Figure S1. Population changes in *Pseudomonas* strains during storage of inoculated fish juice agar (FJA) model substrates at 0 °C (a), 4 °C (b), and 8 °C (c). Results are expressed as means ± stdev of three replicates.
