# Supplementary material for: Growth and Volatile Organic Compound Production of Pseudomonas Fish Spoiler Strains on Fish Juice Agar Model Substrate at Different Temperatures
Source: Microorganisms. 2023 Jan 12;11(1):189. doi: 10.3390/microorganisms11010189 (PMC9861217; doi:10.3390/microorganisms11010189)
Supplement: Supplementary file 1 [file microorganisms-11-00189-s001.zip › Supplementary_final.pdf]

Supplementary material

Table S1: VOCs produced by seven *Pseudomonas* strains in single and mixed cultures in fish juice agar model substrates at 0ºC (a), 4ºC (b) and 8ºC (c). Control is also presented. (please see attached MS excel file)

Figure S1

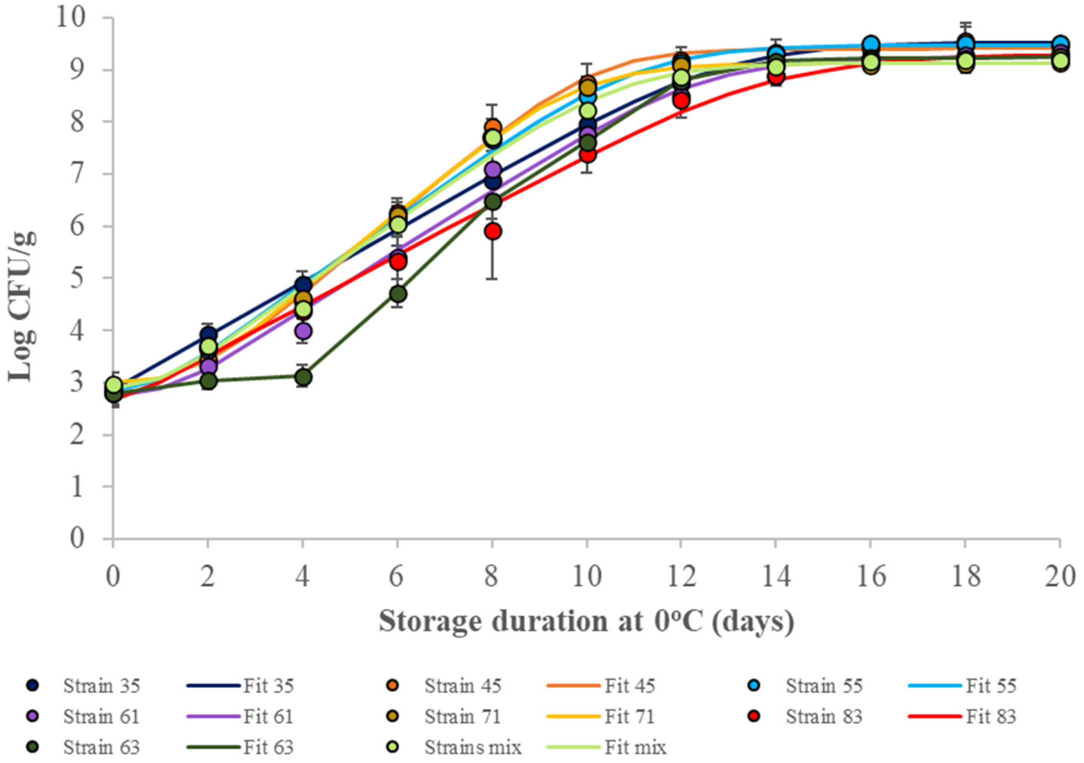

(a)

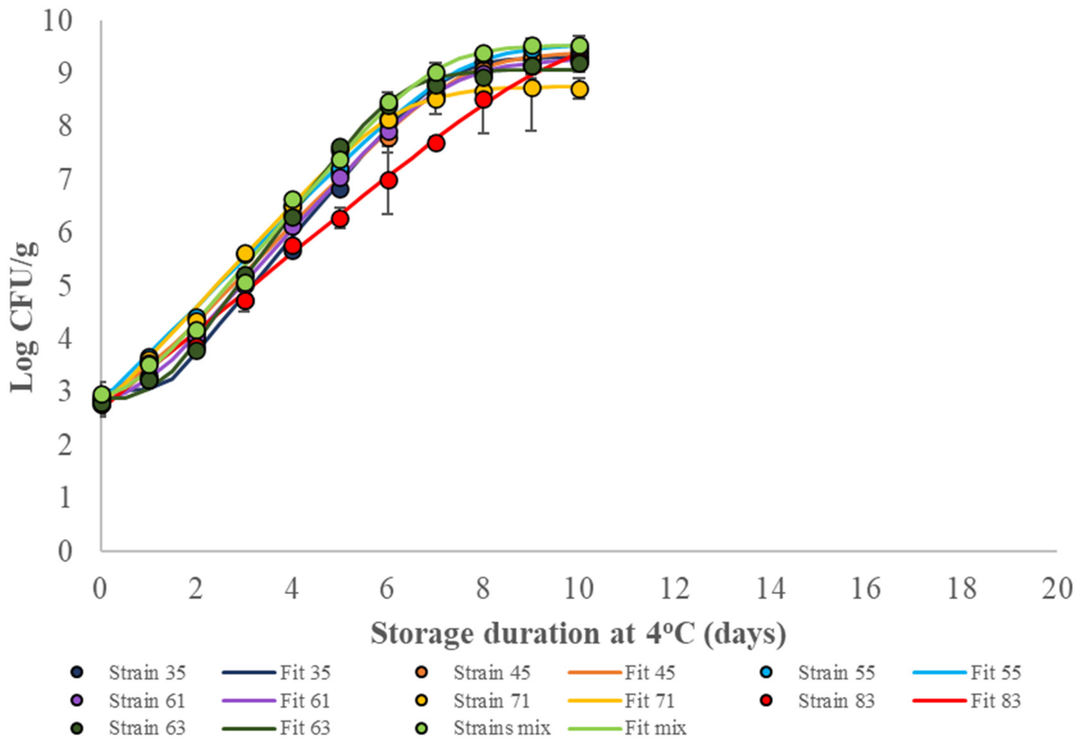

Log CFU/g
Storage duration at 4ºC (days)
Strain 35
Fit 35
Strain 45
Fit 45
Strain 55
Fit 55
Strain 61
Fit 61
Strain 71
Fit 71
Strain 83
Fit 83
Strain 63
Fit 63
Strains mix
Fit mix

(b)

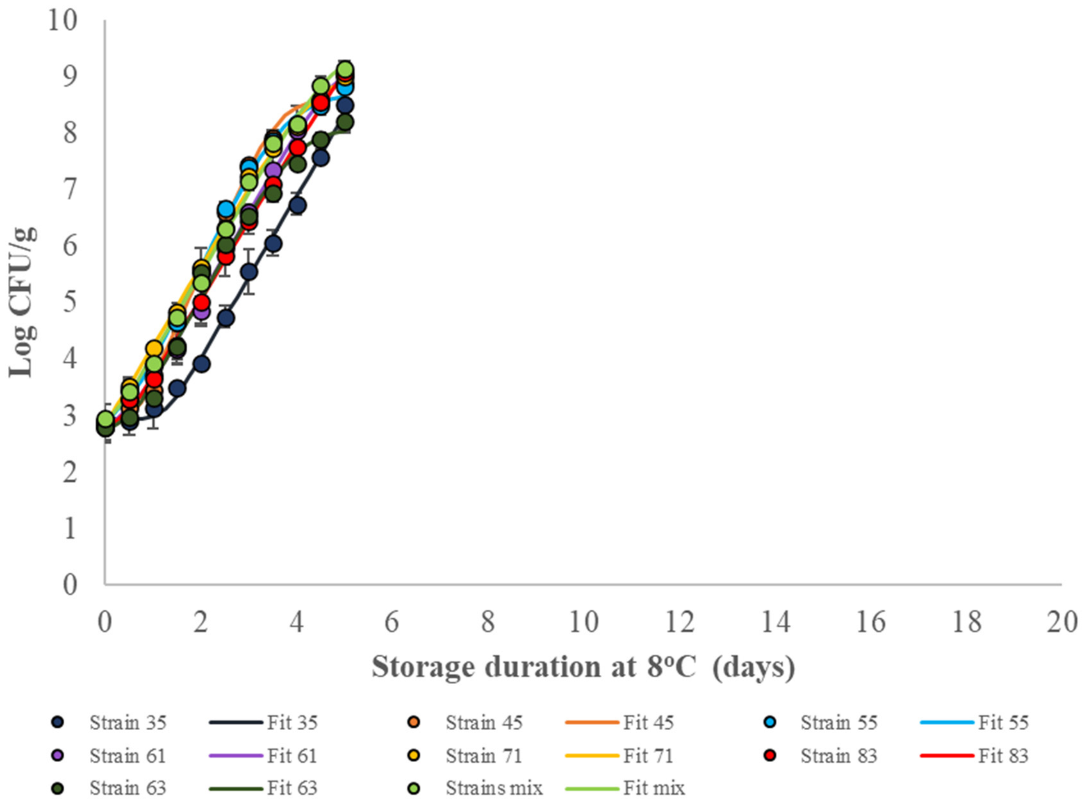

Log CFU/g
Storage duration at 8ºC (days)
Strain 35
Fit 35
Strain 45
Fit 45
Strain 55
Fit 55
Strain 61
Fit 61
Strain 71
Fit 71
Strain 83
Fit 83
Strain 63
Fit 63
Strains mix
Fit mix

(c)

Figure S1. Population changes of *Pseudomonas* strains during storage of inoculated fish juice agar (FJA) model substrates at 0ºC (a), 4ºC (b), and 8ºC (c). Results are expressed as means ± stdev of three replicates.
